# Effects of Mo Particles Addition on the Microstructure and Properties of 316 L Stainless Steels Fabricated by Laser Powder Bed Fusion

**DOI:** 10.3390/ma16134827

**Published:** 2023-07-05

**Authors:** Bolin Li, Shuai Zhang, Shenghai Wang, Li Wang, Yinchuan He, Yaning Cui, Dan Liu, Mingxu Wang

**Affiliations:** 1 School of Mechanical, Electrical & Information Engineering, Shandong University, Weihai 264209, China; 2 Weihai Institute of Industrial Technology, Shandong University, Weihai 264209, China; 3 Weihai Wanfeng Magnesium Industry Science and Technology Development Co., Ltd., Weihai 263200, China

**Keywords:** 316 L stainless steel, laser powder bed fusion, mechanical properties, corrosion resistance

## Abstract

Application of the 316 L stainless steel (SS) is limited by its relatively low wear resistance, insufficient strength, and poor corrosion resistance in special environments. To this end, effects of Mo particles addition on the microstructure, mechanical properties, and corrosion resistance of the laser powder bed fusion (LPBF) 316 L SS are investigated in this study. The results show that the addition of Mo particles from 0 wt.% to 10 wt.% can modify the crystal orientation and improve the strength, wear resistance, and corrosion resistance of LPBF 316 L SSs. Particularly, the LPBF 316 L SS forms a biphasic structure with a similar ratio of α-Fe to γ-Fe with 5 wt.% Mo addition. As a result, the corresponding samples possess both the excellent toughness of austenitic SSs and the high strength and corrosion resistance of ferrite SSs, which reaches a high tensile strength of about 830 MPa, together with a low friction coefficient of 0.421 μ. Since the Mo particles addition is beneficial to increase the content of Cr_2_O_3_ on the samples surface from 13.48% to 22.68%, the corrosion current density of 316 L SS decreases by two orders of magnitude from 569 nA to 6 nA, while the mechanical properties remain favorable. This study is expected to serve as a reference for the preparation of LPBF SSs with excellent integrated performance.

## 1. Introduction

The 316 L stainless steel (SS) has been widely used in machinery, aerospace, and biomedical industry, etc., because of its good ductility, shipping, biomedical, oxidation resistance, and relatively low cost [1]. However, the relatively low strength and localized corrosion susceptibility of the 316 L SS hinders its further structural applications, particularly in the biomedical industry field [2]. Laser powder bed fusion (LPBF) [3] is promising to improve the strength and corrosion resistance of the 316 L SS, as it is able to refine the grains and prevent the formation of detrimental intermetallic phases due to the extremely high cooling rate. Meanwhile, LPBF parts show lower friction resistance compared with the same material produced by a traditional process [4]. Therefore, LPBF 316 L SS is commonly used in many applications, such as heat exchanger, jet engines, and biomedical parts. Nevertheless, the wear resistance, strength, and corrosion resistance of the LPBF 316 L SS cannot meet the requirements in special environments [5,6,7]. Therefore, the performance of LPBF 316 L SSs needs to be further improved.

In recent years, LPBF has been used to prepare coatings to improve the wear resistance and corrosion resistance of 316 L SS due to its layered manufacturing characteristics. Yazici et al. [8] studied the structural, mechanical, and tribological properties of Ti and TiN coatings on 316 L SS by LPBF. However, it is difficult to ensure comprehensive coverage of coatings for complex components in LPBF. Coatings typically occur only on the surface of SS. Due to poor wetting between the coating and the substrate, as well as difficulties in uniform diffusion of elements, metal intermetallic compounds and defects such as cracks often form at the interface.

Furthermore, doping reinforced particle is widely adopted to improve the performance of LPBF 316 L SS due to its ability to directly improve the quality of the formed components without the need for complex coating processes. Han et al. [9] found that the strength of LPBF 316 L SS can be improved by using graphene without affecting ductility. However, the increase in strength is not significant enough to meet the demands of special industrial environments. Some ceramic particles such as TiN [10], TiC [11], SiC [12], have been proven to enhance the strength and wear resistance. The results demonstrated that the improved compressive yield strength mainly originated from the effect of grain boundary strengthening and Orowan strengthening. Song et al. [13] found that Cr_3_C_2_ addition significantly improved the strength of LPBF iron-based materials. However, a large number of cracks and pores resulted in a lower elongation rate of the material. In general, adding ceramic particles can cause a significant spheroidization effect, which has a negative impact on the tensile performance. Meanwhile, the agglomeration of the ceramic particles and the poor interfacial wettability between ceramic particles and the substrate generally causes many defects and cracks, which severely deteriorates the corrosion resistance of the LPBF 316 L SS [14]. Hence, some metal particles are added instead. Fang et al. [15] used a powder mixture of 310 S and 430 SS to prepare γ-Fe composite materials through LPBF. However, the increase in strength of the formed LPBF SS was not significant due to the limited strength of SS materials. Ghayoor et al. [16] improved the mechanical performance of 304 L SS by adding Y. However, the addition of Y to the 304 L matrix resulted in the significant balling effect, thus the improvement in strength is not significant. Sun et al. [17] found that the addition of TiAl in LPBF 316 L SS led to an improvement in the yield strength and ultimate tensile strength. This improvement can be attributed to the refined recrystallized grain structure and the development of a high density of γ-TiAl nanoparticles, which effectively act as barriers to dislocation motion. However, due to the lighter weight of TiAl, segregation occurs at the bottom of the molten pool, resulting in a less significant increase in strength. In comparison with compounds, single-element particles have better electrical conductivity, single structure, and unexpected combinatorial properties. Quan et al. [18] found both electrical conductivity and corrosion resistance of the LPBF 316 L SS were improved after the Ag addition. However, due to the weak interfacial bonding, Ag aggregations tended to split away from the 316 L SS, resulting in the formation of pores. Therefore, the improvement in corrosion resistance is not significant. Yin et al. [19] found that the addition of W particles can improve the mechanical properties of the 316 L SS. However, the addition of W made it difficult to uniformly passivated films form on the surface of 316 L SS which leads to less significant improvement in corrosion resistance. Zhang et al. [20] found that the strength, hardness, and corrosion resistance of LPBF 316 L SS can be improved by the addition of iron-based amorphous alloys. The enhancements of properties are contributed to the oxygen purification of Y element and solid solution of Co and Mo elements, as well as the grain refinement strengthen with the introduction of the amorphous alloy. However, due to the high proportion of Fe in the amorphous material, the improvement in corrosion resistance is not significant. Therefore, we urgently need to find one more kind of reinforcing particles to effectively improve the comprehensive performance of LPBF 316 L SS.

Mo shows high mechanical strength and thermal conductivity [21,22], and the concentration of Mo cations can range from 1 to 20 at.% in passive films [23], indicating Mo particles are promising to improve the performance of the LPBF 316 L SS. However, the impact of Mo’s addition on the strength, wear resistance, and corrosion resistance of LPBF 316 L SS is not clear. In this work, effects of the Mo-particle content on the crystallographic orientation, mechanical properties, tribological properties, and corrosion resistance of the LPBF 316 L SS are systematically investigated. The results highlight the capability of processing stainless steel materials with excellent performance via LPBF to circumvent the harsh requirements on traditional materials.

## 2. Material and Methods

### 2.1. LPBF Facility and Printing Procedure

**Figure 1:**
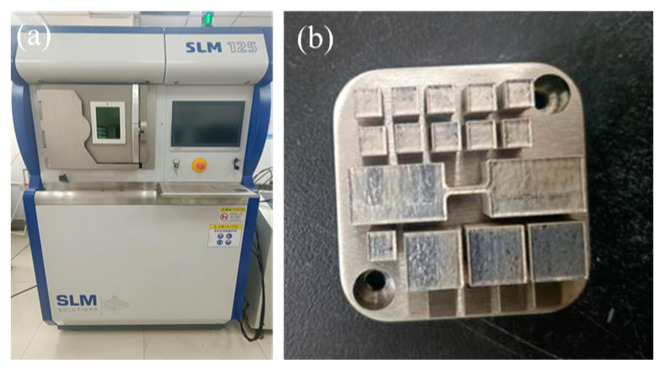
(**a**) LPBF device, (**b**) LPBF sample.

The gas-atomized 316 L SS powders with D_50_ = 39 μm (Sandvik International Trading, Sandviken, Sweden) and Mo powders with D_50_ = 7 μm (Avimetal Am Tech Co, Ltd., Beijing, China) were mixed for 6 h by a three-dimensional motion mixing machine (GH-17L, Nan Fang Powder Equipment Factory, Wenling, China) to ensure they were fully mixed. The particle size distribution of the powders was tested using a laser particle size analyzer (Bettersize2600, Bettersize Instruments Ltd., Dandong, China). In order to systematically investigate the effect of excessive Mo addition on LPBF 316 L SS, specimens with 0, 3, 5, 7, and 10 wt.% Mo addition prepared by the SLM 125 HL (SLM Solutions Inc., Lubeck, Germany) were named as S0, S3, S5, S7, and S10, respectively. The LPBF device and LPBF sample are shown in Figure 1. The LPBF process was performed with oxygen content below 200 ppm. Based on a series of preliminary experiments, the samples were manufactured with a set of LPBF processing optimal parameters listed in Table 1. Moreover, the laser scanning angle between adjacent layers is set to be 33°. Moreover, the substrate is preheated to 120 ℃ to reduce thermal stress and ensure good mechanical stability.

### 2.2. Microstructural Observation

The scanning electron microscopy (SEM; Nova NanoSEM450, FEI Sirion, Hillsboro, OR, USA) was used to observe the microstructure, while phase identification was performed by X-ray diffraction (XRD; Rigaku, Tokyo, Japan). Moreover, compositions of the passive films were analyzed by the X-ray photoelectron spectroscopy (XPS; ESCALAB 250Xi spectrometer, Thermo Scientific Co. Ltd., Waltham, MA, USA).

### 2.3. Mechanical Properties Testing

**Figure 2:**
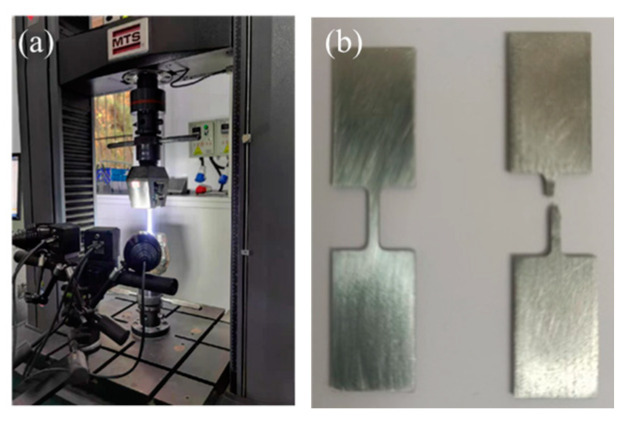
(**a**) Universal testing machine, (**b**) the sample for tensile test.

**Figure 3:**
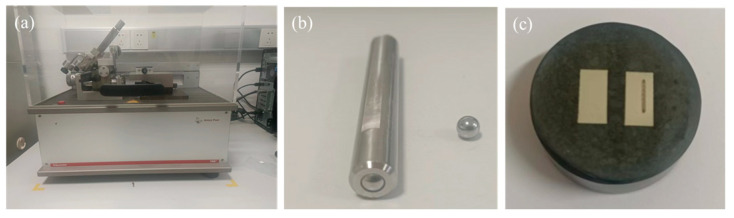
(**a**) Friction and wear tester, (**b**) the grinding ball fixture that holds a ball with a diameter of 6 mm, (**c**) the sample for friction test.

The relative density was measured by an electronic densitometer (ZMD-2, Fangrui Co. Ltd., Shanghai, China) based on the Archimedes principle [24]. Moreover, relative densities of all samples were found to be above 98% in this study. The universal testing machine and tensile sample are shown in Figure 2. The mechanical properties were measured through tensile test running under the universal testing machine (Exceed E45, MTS systems Co. Ltd., Shenzhen, China). Moreover, as shown in the Figure 3a, wear tests were carried out using a friction and wear tester (TRB_3_, Anton Paar Co. Ltd., Graz, Austria). As shown in Figure 2b, a linear reciprocating dry friction wear test method was adopted, where the grinding ball with a diameter of 6 mm is used. The worn surfaces were characterized by the optical microscope (OM, VK-X1000, Keyence, Osaka, Japan) and SEM. In addition, the cross-sectional area of wear tracks was determined by the average value of from four measurements long −X, +X, −Y, and +Y directions, respectively. Moreover, the friction experiment is conducted at room temperature, with a load of 2 N and a frequency of 6 Hz.

### 2.4. Electrochemical Testing

**Figure 4:**
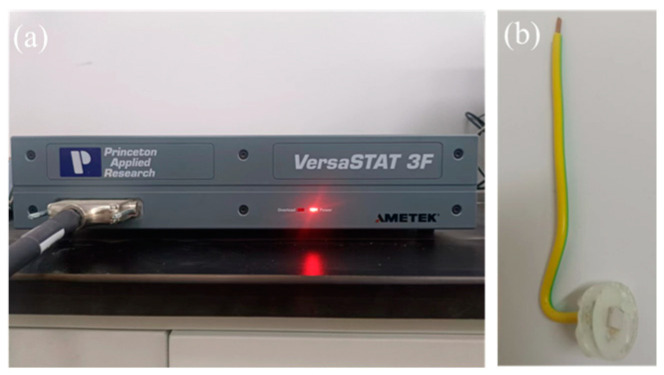
(**a**) Electrochemical station, (**b**) the sample for electrochemical test.

The electrochemical station (VersaSTAT 3F, Ametek Co. Ltd., California, USA) and electrochemical samples were shown in Figure 4. To minimize errors arising from the varying formation times of passivation films, electrochemical samples are prepared concomitantly. To ensure the reproducibility, all the measurements were repeated at least 3 times under 20 ± 2 °C.

## 3. Results and Discussion

### 3.1. Phase Analysis

**Figure 5:**
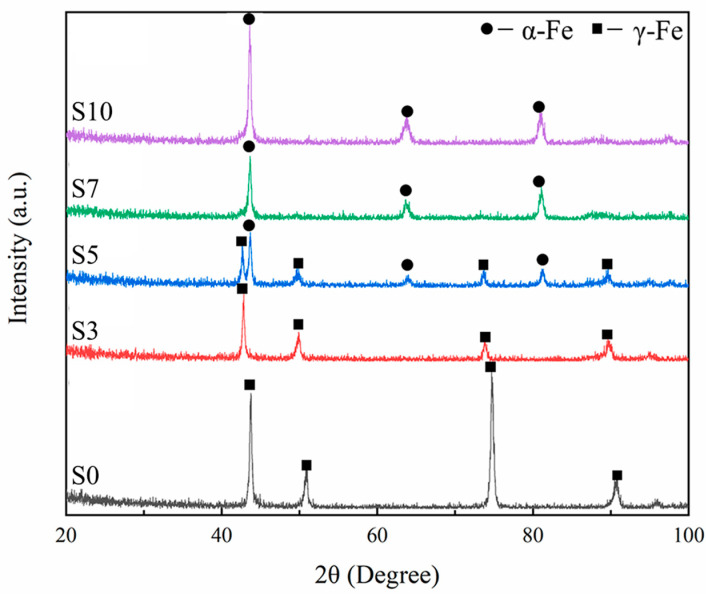
XRD patterns of the LPBF samples.

To identify the phase structure, XRD patterns of the samples are presented in Figure 5. One sees from Figure 5 that with the increasing Mo content, the phase transformation occurs from γ-Fe for S0 to α-Fe for S10. Especially, both phases are contained in S5. The phase transformation can be attributed to the change of the chemical composition and cooling rate [25]. During the solidification process, more MoC_x_ forms and segregates at the grain boundaries due to the strong affinity and the co-segregation between Mo and C [26] with the increasing Mo addition, which results in a reduction in C concentration within the 316 L matrix, and then promotes the generation of α-Fe with lower C than γ-Fe. Meanwhile, the thermal conductivity of Mo (142 W·m^−1^·K^−1^) is about 10 times higher than that of 316 L SS (14 W·m^−1^·K^−1^) [27,28]. The thermal conductivity of the hybrid powder increases with the increasing Mo addition, which results in a higher cooling rate. Furthermore, the high cooling rate leads to the formation of the α-Fe [29]. Moreover, the increasing Mo addition results in the larger lattice distortion of the matrix due to the relatively large atomic diameter, which causes higher tensile stress within the matrix and in turn promotes the stress-induced transformation from γ-Fe to α-Fe [30].

### 3.2. General Microstructure

**Figure 6:**
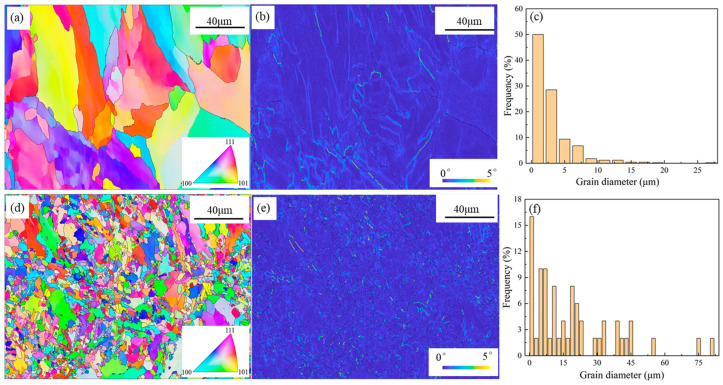
Inverse pole figure (**a**,**d**), Kernel-average-misorientation map (**b**,**e**), and grain size distribution (**e**,**f**) of the LPBF samples. S0: (**a**–**c**), S5: (**d**–**f**).

The duplex SS has been proven to combine the excellent toughness and weldability of γ-Fe with the high strength and chloride corrosion resistance of α-Fe [31]. Thus, the S5 containing the duplex phase is compared with S0 in this work. Moreover, the tissue structures for S0 and S5 are shown in Figure 6. One sees from Figure 6a,d that there are a lot of irregular grains in S0 and S5, which results from the rapid heating/cooling rate of the LPBF technique (>10^5^ K/s). Figure 6b,e shows that the addition of Mo tends to increase the dislocation density of the LPBF 316 L. While the mean grain size is largely decreased from 18 μm for S0 to 3 μm for S5, as shown in Figure 6c,f. The formed preferential Mo_x_C in the molten pool can supply effective heterogeneous nucleation during the solidification process, which results in the grain refinement [32]. Moreover, the higher cooling rate caused by the increasing Mo addition can also play a key role in the grain refinement.

### 3.3. Tensile Properties

**Figure 7:**
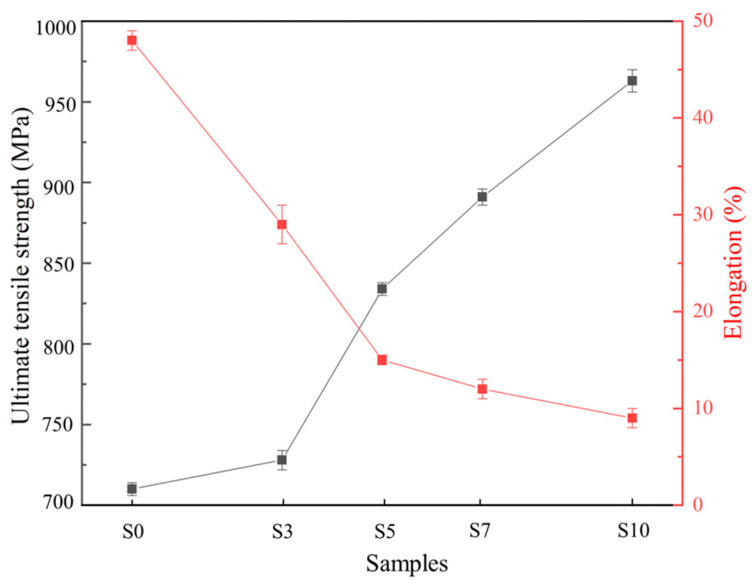
Tensile curves of the LPBF samples.

Tensile tests are conducted to investigate the effect of Mo particles on the mechanical properties of 316 L SS. Tensile properties of the LPBF samples are shown in Figure 7, where one sees that the ultimate tensile strength increases with increasing Mo addition, from 710 MPa for S0 to 963 MPa for S10. Moreover, the significant increase in strength with more than 3 wt.% Mo particles addition can be attributed to the formation of brittle α-Fe. While the insignificant decrease in elongation when the Mo addition exceeds 5 wt.% results from the grain refinement, as it can improve both the strength and plasticity of the material [33]. Considering that S5 has both good plasticity and strength, we believe that S5 shows the best comprehensive tensile properties in this study.

### 3.4. Fracture Surface

**Figure 8:**
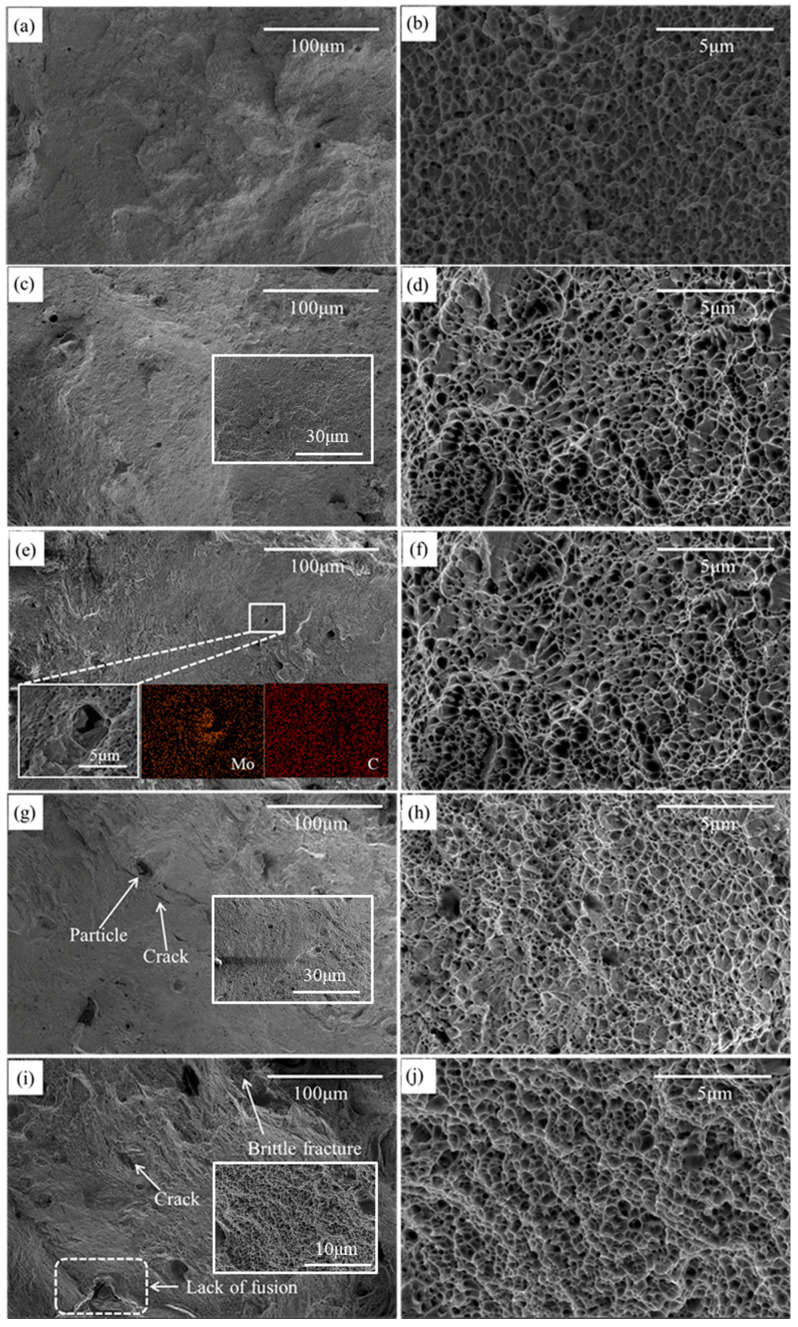
Tensile fracture topography. (**a**,**b**) S0, (**c**,**d**) S3, (**e**,**f**) S5, (**g**,**h**) S7, and (**i**,**j**) S10.

To investigate fracture mechanism of S0, S3, S5, S7, and S10, the tensile fracture topography is shown in Figure 8. One sees from Figure 8 that S0, S3, S5, and S7 show small and deep dimples, indicating that all samples exhibit typical characteristics of ductile fracture. Moreover, the traces of brittle fracture for S10 can be seen in in Figure 8i, illustrating the combination of ductile fracture and brittle fracture. Moreover, the worsened plasticity can be attributed to the increasing number of defects. As shown in the insert of Figure 8e, several partially molten steel particles are found on the fracture surface. The surrounding surface of these particles did not show any pits. These partially molten areas formed at the interface between two layers are believed to represent a fundamental cause of the scattering in elongation to failure and premature fractures detected in tensile samples [34,35]. As shown in Figure 8g,f with the increasing Mo particle addition, the cracks easily propagate from the defects when the tips reach them, ultimately leading to sample fracture [36]. The addition of Mo can effectively refine the grain size. Moreover, the increase in the number of grains is advantageous for dispersing plastic deformation caused by external forces, thereby reducing stress concentration. In addition, the finer the grains, the larger the grain boundary area, which is less conducive to crack propagation. Therefore, the decrease in plasticity is not significant when the Mo addition exceeds 5 wt.%. Moreover, the enhancement of strength stems from the synergistic influence of fine grain strengthening, dislocation strengthening and solid solution strengthening. The solid solution of Mo atoms with large atomic radius in austenite leads to the precipitation of Ni and Cr elements at grain boundaries, which causes more pinning points, hinders the expansion of grain boundaries and refines grains. Thus, S10 should have a higher strength. Furthermore, similar element distribution is found for Mo and C as shown in the inset of Figure 8e, which confirmed the existence of MoC_x_ to some extent.

### 3.5. Tribological Performance

**Figure 9:**
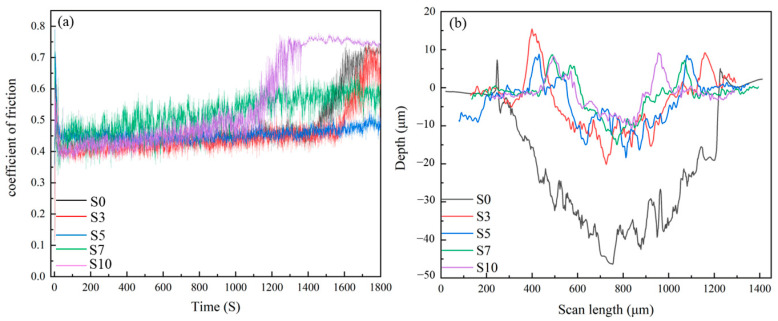
(**a**) The relationship between the coefficient of friction and time for LPBF samples, (**b**) the sectional profile of wear tracks of LPBF samples.

To reveal the influence of Mo content on the wear resistance of 316 L SS, the relationship between the coefficient of friction and time and profiles of the cross section of the wear track for the samples are shown in Figure 9a,b. Moreover, the corresponding tribological characteristics of LPBF samples are listed in Table 2. One sees from Figure 9 and Table 2 that both the width and depth of the abrasion marks decrease as the Mo content increases, indicating that the addition of Mo can effectively improve the wear resistance. While the friction coefficient of the samples decreases firstly and then increases with the increasing Mo addition. The reduction in the friction coefficient of S3 and S5 can be mainly attributed to the enhancement of both the solid solution strengthening and refinement strengthening with the increasing Mo. Moreover, the increasing friction coefficient from S7 and S10 can be ascribed to the cracks revealed in Figure 8e,i. The emergence of flaws elevates the surface roughness, resulting in a certain escalation in the friction coefficient of S7 and S10. In contrast, S5 has the lowest coefficient of friction, while S10 has the lowest wear rate coefficient.

### 3.6. Worn Morphology

**Figure 10:**
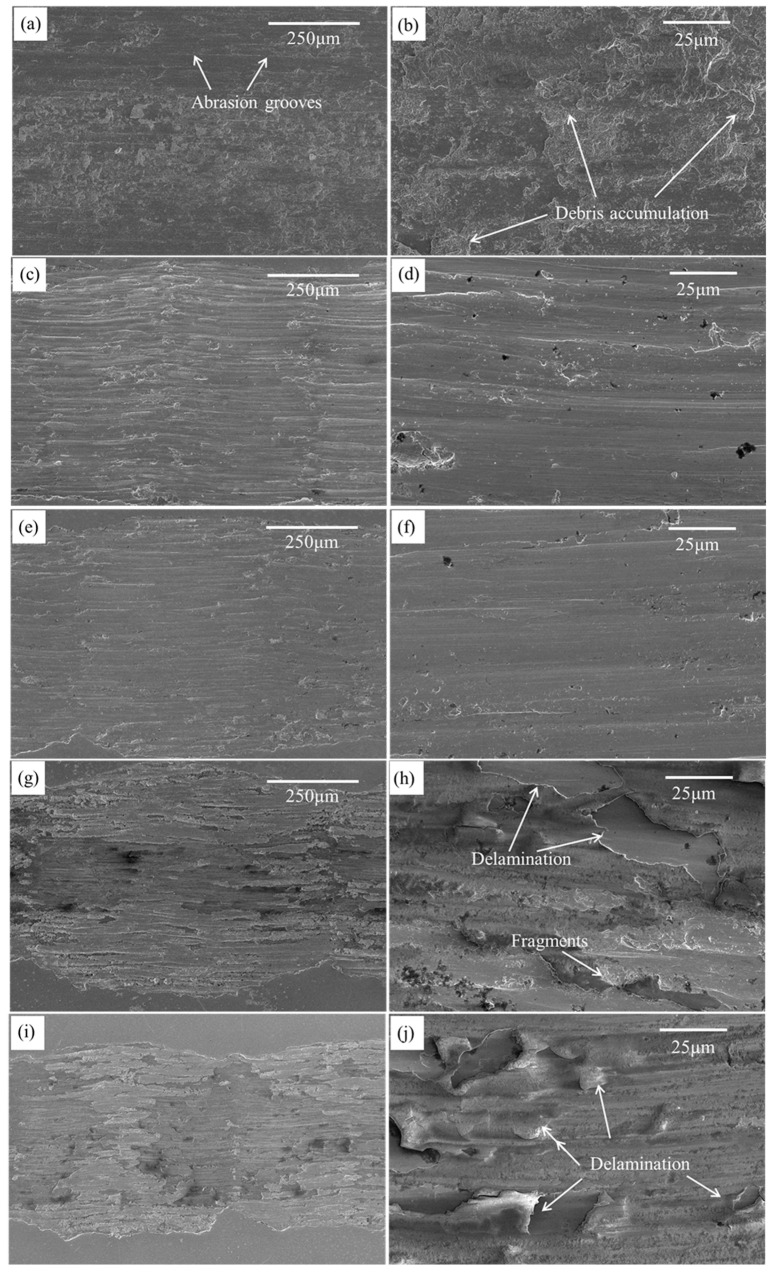
SEM images of wear tracks for the LPBF samples. (**a**,**b**) S0, (**c**,**d**) S3, (**e**,**f**) S5, (**g**,**h**) S7, (**i**,**j**) S10.

To further analyze the wear mechanism, wear patterns are obtained as shown in Figure 10. One sees from Figure 10 that the stripped worn debris is plowed along the direction of the slide, forming grooves of varying depths. The stripped material is crushed by the matrix and the grinding ball in the process of wear, forming the three-body friction of matrix, wear debris, and grinding ball. Moreover, the wear surfaces of specimen S0 shows a deep wear track with severe delamination. However, the worn surfaces of S3 and S5 are relatively smooth, with shallow grooves and covered by a thick uniform tribo-layer. While significant wear surface peeling can be found for S7 and S10. As shown in Figure 10b, the predominant forms of wear observed in S0 are adhesive and abrasive wear, along with oxidative wear resulting from increased temperatures during friction [37]. These types of wear are largely attributed to the low hardness of the material. In contrast, the wear morphology of S3 and S5 is characterized by mild abrasive plowing and oxidative wear, as illustrated in Figure 10d,f. The lack of discernible adhesive wear morphology observed in samples S3 and S5 can be attributed to the synergistic impact of solid solution strengthening, fine grain strengthening, and dislocation strengthening. As shown in Figure 9g–j, the surface peeling of S7 and S10 occurs during high-speed friction, which can be attributed to the inadequate welding properties and increased content of the brittleness α-Fe with the increasing Mo addition. The rough surface produced during the wear process resulted in an increase in the friction coefficient of S7 and S10.

### 3.7. Electrochemical Results

**Figure 11:**
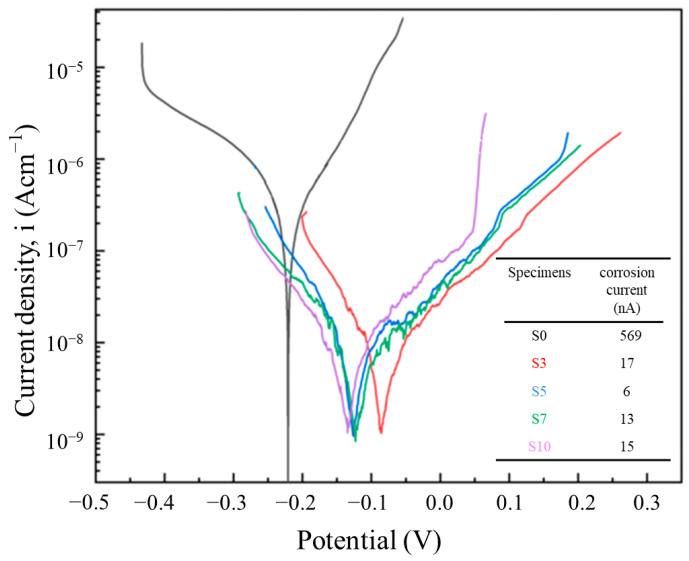
Potentiodynamic polarization curves of LPBF samples.

In order to evaluate the localized corrosion susceptibility of LPBF samples in a NaCl environment, potentiodynamic polarization curves are shown in Figure 11. As depicted in Figure 11, the addition of Mo can significantly enhance the resistance of 316 L SS against pitting corrosion. However, the corrosion current density first decreases and then increases slightly with the increasing Mo content. Moreover, the S5 shows the best corrosion resistance in this study, whose corrosion current density is about two orders of magnitude lower than that of S0. Meanwhile, there is almost no overlap between the cathode and anode branches for all the samples, indicating that the addition of Mo has a great impact on both the cathode and anode reaction. Researchers found that the corrosion current density is closely related to the composition of the surface passivation film [38]. Therefore, it can be inferred that the addition of Mo particles may improve the corrosion resistance by changing the composition of the passivation film.

### 3.8. Passive Film Characterization

**Figure 12:**
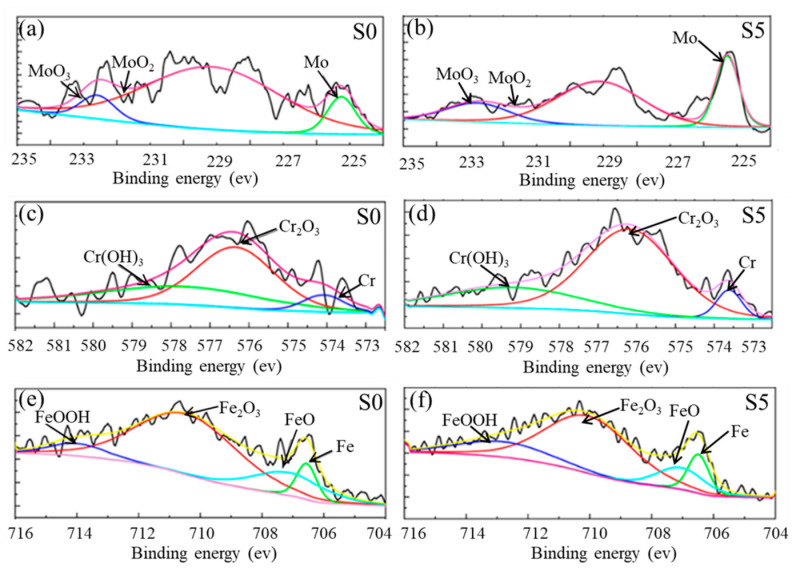
The detailed XPS spectra of Mo 3d3/2 (**a**,**b**), Cr 2p3/2 (**c**,**d**), and Fe 2p3/2 (**e**,**f**) of the passive films formed on S0 and S5.

**Figure 13:**
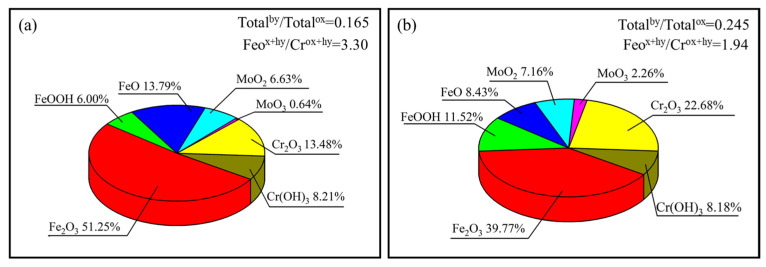
Cationic fraction in the passive film formed on S0 (**a**) and S5 (**b**).

Composition and structure are two key issues in passivated films, which have an essential impact on the corrosion properties of samples [39,40]. To investigate the influence of Mo particles on the surface passivation film, the XPS spectra and cationic fraction of S0 and S5 are shown in Figure 12 and Figure 13. Moreover, based on the integral areas of the corresponding peak, the oxide species in the passive film are calculated. One sees from Figure 12 that for both S0 and S5, the passive films are primarily composed of the oxides and hydroxides of Fe and Cr. As verified in Figure 12, there are four main peaks in the electronic orbit of Fe_2_p_3/2_, which correspond to the Fe (706.5 eV ± 0.1 eV), FeO (707.3 eV ± 0.1 eV), Fe_2_O_3_ (710.8 eV ± 0.1 eV), and FeOOH (714.2 eV ± 0.1 eV), respectively. Due to the rapid dissolution of Fe compounds in corrosion solution, the dissolution of passive films is quick when a large number of Fe compounds exist in passive films, leading to the poor corrosion resistance of passive films [41]. The addition of Mo reduces the proportion of iron compounds in the passivation film and improves corrosion resistance. As illustrated in Figure 12, there are three main peaks in the electronic orbit of Cr_2_p_3/2_, namely Cr (574.1 eV ± 0.1 eV), Cr_2_O_3_ (576.3 eV ± 0.1 eV), and Cr(OH)_3_ (577.1 eV ± 0.1 eV). Cr_2_O_3_ and Cr(OH)_3_ are the typical compounds of Cr in passive films of stainless steels. Compared with the Cr hydroxide, Cr_2_O_3_ is the main compound affecting the compactness and corrosion resistance of passive film [42]. Moreover, it can be found from Figure 13 that Mo addition can effectively improve the content of Cr_2_O_3_ in passivated films, which is consistent with the reported results [43]. This means that S5 has a denser passivation film and better corrosion resistance. Simultaneously, the inclusion of Mo can enhance the MoO_x_ content within the passivation film [44], which can effectively inhibit the dissolution of Cr_2_O_3_. The ratio of the total hydroxides and total oxides in the passive films formed on S0 (approximately 0.165) is smaller than that in the passive film formed on S5 (nearly 0.245), which indicates that the passive film of S0 has a lower content of hydroxides than S5. According to the bilayer theory of passive film [45], Cr_2_O_3_ is the primary constitute of the inner layer, while unstable oxides and hydroxide of Fe and Cr(OH)_3_ are mainly distributed in the outer layer. It can be thus concluded that the Mo addition can promote the stable formation of the inner passivation film, which leads to improved charge transfer resistance and hinders further corrosion.

## 4. Conclusions

In order to improve the performance of 316 L SS and to serve guidance for the development of advanced materials, we fabricated a superior performing 316 L SS by adding Mo particles from 0 to 10 wt.% via the LPBF technique. Moreover, the corresponding microstructure, tensile properties, wear performance, and corrosion resistance of the samples have been analyzed. The conclusions are as follows:

1. With the increasing Mo particles addition, phase transformation occurs from γ-Fe for S0 to α-Fe for S10. Especially, both the phases are contained in S5. Moreover, the phase transformation can be attributed to the formation of MoC_x_ and the increasing cooling rate as well as the increasing internal stress caused by Mo addition.
2. With the increasing Mo particles addition, the ultimate tensile strength of the LPBF 316 L SS increases monotonically, while the friction coefficient decreases firstly till 5 wt.% Mo addition and then increases. Moreover, S5 shows the best comprehensive mechanical performance in this study, which can be attributed to the synergistic impact of solid solution strengthening, fine grain strengthening, and dislocation strengthening, as well as the relatively low content of defects caused by Mo addition.
3. The addition of Mo can improve the corrosion resistance by increasing the content of Cr_2_O_3_ in the passivated film. Moreover, a low corrosion current up to 6 nA is achieved for S5, which has two orders of magnitude lower than that of S0. However, too much Mo particles (e.g., >5 wt.%) leads to the increase in defects. Therefore, S5 shows the best corrosion resistance in this study.

## Figures and Tables

**Table 1:** The LPBF processing parameters of samples.

Samples	Power (W)	Scanning Speed (mm/s)	Layer Thickness (mm)	Laser Energy (J/mm^3^)
S0	200	800	0.03	69
S3	215	800	0.03	75
S5	225	800	0.03	78
S7	235	800	0.03	82
S10	250	800	0.03	87

**Table 2:** Tribological properties of samples.

Specimens	Frictions Coefficient (μ)	Wear Depth (μm)	Wear Width (μm)	Wear Track Area (μm^2^)
S0	0.473	47 ± 2.4	1018 ± 42	33,839 ± 342
S3	0.432	21 ± 1.7	820 ± 29	10,915 ± 247
S5	0.421	18 ± 1.6	650 ± 33	9592 ± 153
S7	0.479	14 ± 2.1	585 ± 27	6738 ± 324
S10	0.493	9 ± 1.4	473 ± 35	5382 ± 173

## Data Availability

All data included in this study are available upon request by contact with the corresponding author.
